# Interactions Between the Circadian Clock and Heme Oxygenase in the Retina of *Drosophila melanogaster*

**DOI:** 10.1007/s12035-016-0026-9

**Published:** 2016-08-13

**Authors:** Milena Damulewicz, Agnieszka Loboda, Alicja Jozkowicz, Jozef Dulak, Elzbieta Pyza

**Affiliations:** 1 Department of Cell Biology and Imaging, Faculty of Biology and Earth Sciences, Jagiellonian University, 30-387 Krakow, Poland; 2 Department of Medical Biotechnology, Faculty of Biochemistry, Biophysics and Biotechnology, Jagiellonian University, 30-387 Krakow, Poland

**Keywords:** *Drosophila*, Circadian clock, Heme oxygenase, Retina, Carbon monoxide, Nitric oxide

## Abstract

The *Drosophila* retina has an autonomous peripheral circadian clock in which the expression of the gene encoding heme oxygenase (HO) is under circadian control with the *ho* mRNA peaking at the beginning of the day and in the middle of the night. The function of HO in the retina is unknown, but we observed that it regulates the circadian clock and protects photoreceptors against DNA damage. The decline in HO level increases and decreases the expression of the canonical clock genes *period* (*per*) and *Clock* (*Clk*), respectively. The opposite result was observed after increasing HO expression. Among three products of HO activity—carbon monoxide (CO), ferrous ions, and biliverdin—the latter has no effect on *per* and *Clk* expressions, but CO exerts the same effect as the increase of *ho* expression. This suggests that HO action on the clock is mediated by CO, which may affect *Clk* expression during the day and the level of *per* expression. While *ho* expression is not stimulated by nitric oxide (NO), NO has the same effect on the clock as HO, increasing *Clk* expression and decreasing the expression of *per*.

**Electronic supplementary material:**

The online version of this article (doi:10.1007/s12035-016-0026-9) contains supplementary material, which is available to authorized users.

## Introduction

Heme oxygenase (HO) is an enzyme that degrades heme to carbon monoxide (CO), ferrous ions, and biliverdin. In mammals, there are two isoforms of HO encoded by two different genes, inducible HO-1 and constitutive HO-2. Although they differ in distribution and functions, they both act as cytoprotective and anti-apoptotic agents in an organism, by scavenging reactive oxygen species (ROS; reviewed in [1]). In *Drosophila melanogaster*, there is only one gene encoding HO [2] that plays an important role in development [3] and in controlling the signaling pathway of DNA damage [4]. Any other functions of HO in insects are still unknown.

In the suprachiasmatic nuclei (SCN), the site of the mammalian circadian pacemaker for behavioral rhythms, HO activity changes during the day, reaching the maximum during the night. This pattern is maintained in constant darkness, suggesting that the activity of HO is controlled by the circadian clock [5]. Moreover, changes in the heme level influence the expression of clock genes [6,7] as well as the degradation of the clock protein PERIOD 2 (PER2) [7]. This specific function of heme is tissue-dependent [8]. In *D. melanogaster*, cyclic expression of the *ho* gene has been found in whole head homogenates [9], but nothing is known about the role of HO in the circadian system.

In *Drosophila*, as in all other animals, apart from the central pacemaker, there are peripheral clocks, one of the most studied being located in the retina. This oscillator generates cyclic processes in the retina and partly in the lamina, the first neuropil of the optic lobe [10–12]. The molecular mechanism of the retina clock is similar to that of the pacemaker and is based on several interlocked feedback loops [10,13]. In the main negative feedback loop, PERIOD (PER) and TIMLESS (TIM) proteins inhibit transcription of their own genes *per* and *tim*, the core clock genes. The expression of *per* and *tim* is regulated by the transcription factors CLOCK (CLK) and CYCLE (CYC) that, late in the evening, dimerize and enter the nucleus, binding to the regulatory E-box sequences in the promoters of *per* and *tim*, inducing their expression. At the same time, CLK and CYC control the expression of clock-controlled genes, and one of them could conceivably be *ho*. In turn, PER and TIM proteins dimerize late at night and are transported to the nucleus, where they bind to CLK/CYC and inhibit their activity, thereby halting the transcription of *per* and *tim*. In contrast to the pacemaker, CRYPTOCHROME, a circadian photoreceptor, acts as a repressor in the retinal clock and inhibits CLK activity [14].

Since HO may play a cytoprotective function and the retina is a site of intense physiological processes of phototransduction that generates high levels of ROS [15], we examined whether this protein is cyclically expressed in the retina, in parallel to circadian rhythms in the fly’s visual system [16], and whether HO influences the retinal clock and protects photoreceptors against degeneration. We found that *ho* gene expression is controlled by the circadian clock and HO also regulates the clock of *D. melanogaster*. Moreover, HO reduces DNA damage after exposure to UV and white light.

## Materials and Methods

### Animals

We used the following strains of *D. melanogaster*: wild-type Canton S, *Clk* ^*Jrk*^ (mutant in which premature stop codon disrupts the activation domain of the CLOCK protein) [17]; *cyc* ^*0*^ (recessive null mutant of the *cycle* gen) [18]; *per* ^*01*^ (null mutant of the *period* gene) [19]; *Rh1*-*Gal4* (strain which expresses GAL4 under the control of the *rhodopsin1* promoter) [20]; *repo*-GAL4 (strain which expresses GAL4 under the control of the *repo* (*reversed polarity*) promoter–glial cell marker); UAS-*gfp*, UAS-*hoRNAi* (strain which expresses dsRNA for *ho* gene under the control of UAS sequence) [3]; and UAS-*Valium10*-*gfp* (control for RNAi flies) [21]. Flies were maintained under 12 h of light and 12 h of darkness (LD12:12) conditions or in constant darkness (DD) and at a constant temperature of 24 °C.

### In Vitro Experiments

For in vitro experiments, we used Schneider 2 cells (S2; Invitrogen) derived from a primary culture of late-stage (20–24 h old) *D. melanogaster* embryos [22]. Cells were cultured in Schneider’s medium (Invitrogen) with 10 % fetal bovine serum (Invitrogen) and 100 U/ml penicillin/100 μg/ml streptomycin (MP Biomedicals) at 25 °C.

### Experimental Procedure

To detect cyclic expression of *ho* in the retina, we analyzed *ho* messenger RNA (mRNA) level at five time points in LD12:12 and DD conditions: 1 h after the beginning of the day (Zeitgeber Time, ZT1)/the subjective day (Circadian Time, CT1); 4 h after the beginning of the day (ZT4)/the subjective day (CT4); 1 h after the beginning of the night (ZT13)/the subjective night (CT13); 4 h after the beginning of the night (ZT16)/the subjective night (CT16); and 8 h after the beginning of the night (ZT20)/the subjective night (CT20). ZT0 indicates the beginning of the light/day phase and ZT12 stands for the beginning of the night/dark phase in LD12:12, whereas CT0 and CT12 stand for the beginning of the subjective day and the beginning of the subjective night, respectively, in DD. It has been reported that significant differences in the expression level of many clock-controlled genes occur at these time points [10,12]. In addition, we examined *ho* expression profile exclusively in glial cells which were sorted out of the brain according to the methods published previously [10]. For this experiment, we used *repo>gfp* and *repo>hoRNAi*,*gfp* strains and examined *ho* mRNA level at ZT1, ZT4, ZT13, and ZT16.

To study the effects of HO on the clock in the retina, we applied several chemicals and examined the expression of clock genes *per* and *Clk*. Flies were starved for 12 h with water available ad libitum; next, they were fed for 12 h (until decapitation) with 6 % glucose solution supplemented with: 100 μM of hemin chloride (Calbiochem), a heme oxygenase activator; 100 μM of tin protoporphyrin IX (SnPPIX, Frontier Scientific), a heme oxygenase inhibitor; 500 μM of carbon monoxide-releasing molecule 2 (CORM-2, Sigma-Aldrich), a carbon monoxide (CO) donor; 500 μM of *S*-nitroso-*N*-acetylpenicillamine (SNAP, Sigma-Aldrich), a nitric oxide (NO) donor; or 500 μM of biliverdin dihydrochloride (BV, MP Biomedicals). Control flies were fed with 6 % glucose solution with addition of NaOH (solvent for hemin, SnPPIX, SNAP, and BV). In the case of the experiment with CORM-2, control flies were fed with 500 μM of the inactive compound (iCORM) which does not release CO (prepared in DMSO, similarly to CORM-2, but left overnight in room temperature to liberate CO).

To establish the proper concentration of SNAP for experiments, we examined the ROS level in the brain of *Drosophila* after SNAP application. It has been found that the application of SNAP specifically increases ROS production in cells [23]. First, flies were starved for 12 h and then fed with 100, 250, or 500 μM of SNAP for 12 h. Then, brains were isolated and treated with trypsin/EDTA for 1 h at 37 °C. The obtained cells were centrifuged for 10 min at 10,000×*g* and then resuspended and incubated in phosphate-buffered saline PBS with 10 μM ROS Detection Reagents (Invitrogen) for 1 h at 37 °C. Next, they were centrifuged again, resuspended in PBS, and analyzed by flow cytometry. The cells obtained from flies not fed with SNAP served as a control. As a negative control, cells without ROS dye treatment were used.

To determine DNA damage in the retina photoreceptors after light exposure and different HO levels in cells, flies were kept in DD for 5 days and starved for 6 h; water was available ad libitum. Next, they were fed for 6 h (until decapitation) with 6 % glucose in water supplemented with HO activator, 100 μM hemin chloride (Calbiochem), or HO inhibitor, 100 μM tin protoporphyrin IX (SnPPIX, Frontier Scientific). The control group was fed only with glucose. All groups of flies were exposed to 1 h UV light pulse (100 lx) at CT0 and then to 3 h white light (1500 lx) emitted from UV and white light UVECX26 and TFX53WL transluminators (Vilbert Lourmat), respectively. After that they were decapitated and fixed in 4 % paraformaldehyde. Cryosections were prepared and immunodetection with anti-8-hydroxyguanosine primary antibody (1:500, overnight), which labels oxidative DNA damages, was carried out. On the following day of the procedure, HRP/DAB (ABC) detection kit was used according to the manufacturer’s protocol (Abcam). Negative control was prepared without the primary antibody. In positive control, heads were decapitated and incubated for 4 h in Schneider’s medium supplemented with etoposide, a cytotoxic compound. We also used two different control groups: the first group was fed with glucose and exposed to UV and white light and the second one was collected in DD.

Clock gene expression was also studied in S2 cells which were cultured in Schneider’s medium supplemented with 100 μM of hemin, SnPPIX, CORM-2, BV, or SNAP for 6 h. Time of exposure and the concentration of the chemicals listed above were established during our preliminary study. Control cells were treated with the solvent or with iCORM. S2 cells do not have circadian clocks, but express some clock and clock-controlled genes and can be used to study certain aspects of the circadian clock functioning [24].

### RNA Isolation and qPCR

Males, 7 days old, were decapitated at ZT1, ZT4, ZT13, and ZT16 under LD12:12 or at CT1, CT4, CT13, and CT16 under DD. Heads were fixed in 100 % ethanol for 2 h and retinas were isolated. Approximately 30 flies were used for each time point, and each experiment was repeated at least three times.

Total RNA isolation was performed using TriReagent (MRC Inc.) according to the manufacturer’s protocol. The complementary DNA (cDNA) for polymerase chain reaction (PCR) amplification was prepared from 1 μg total RNA using Superscript II reverse transcriptase (Life Technologies) according to the provider’s instruction. Gene expression was examined using TaqMan Gene Expression Assays labeled with 6′-FAM (Applied Biosystems) and 7500 Fast Real-Time PCR System (Applied Biosystems). The following genes were examined: *Ribosomal protein 32* (*rpl32*, Dm02151827_g1) as a reference gene; *period* (*per*, Dm01843683_g1); and *Clock* (*Clk*, Dm01795381_g1). cDNA, diluted 1:10, was used for quantitative PCR. Each experiment was repeated at least three times. The expression of *ho* gene was examined using SYBR Green Master Mix (Applied Biosystems) and the following primers: forward primer: 5′-ACCATTTGCCCGCCGGGATG; reverse primer: 5′-AGTGCGACGGCCAGCTTCCT; for *rpl32*: forward primer: 5′-AGAAGCGCAAGGAGATTGTC; reverse primer: 5′-ATGGTGCTGCTATCCCAATC. Product specificity was assessed by melting curve analysis, and selected samples were run on 1 % agarose gels for size assessment.

Data were collected as raw *C*_T_ values and analyzed using the 2^−ΔΔ*C*T^ method. Gene expression was normalized on an arbitrary scale with control (or ZT1) as 1.0.

### Statistical Analysis

Statistical analysis was performed using non-parametric analysis of variance (ANOVA) Kruskal–Wallis test and then Tukey’s test or non-parametric Mann–Whitney test to compare differences between groups fixed at different time points or between experimental and control groups. Statistica 7.0 software was used for analysis, and statistically significant differences were at *p* < 0.05.

## Results

### Expression of *ho* in LD12:12 and DD

**Fig. 1:**
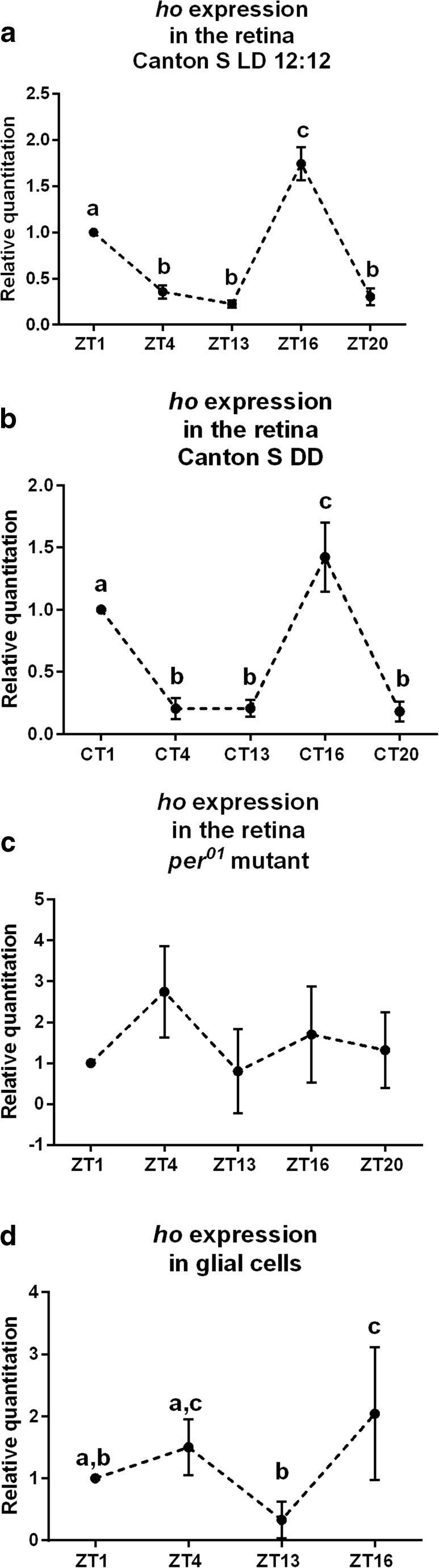
Expression of *ho* gene in the retina of wild-type *D. melanogaster* at four time points in LD12:12 (**a**) and DD conditions (**b**) and in *per* ^*01*^ mutant (**c**) in LD12:12 as well as in glial cells (**d**). Statistically significant differences between time points are marked with *different letters. Graph* presents relative quantitation of steady-state mRNA level ± SD; data are normalized to ZT1 in LD12:12 or CT1 in DD (value = 1.0). Non-parametric Kruskal–Wallis test, parametric ANOVA Tukey’s test: *p* < 0.05

The examination of *ho* mRNA level at five time points during the day in the retina showed that *ho* expression changes during the day and night in LD12:12 (Fig. 1a), with two peaks at ZT1 and ZT16. The differences between ZT1, ZT16, and other time points were statistically significant. This bimodal pattern was maintained in constant darkness (DD; Fig. 1b) and the rhythm was abolished in *per* ^*0*^ (Fig. 1c), indicating that *ho* expression is controlled by a circadian clock. The examination of *ho* expression pattern in glial cells showed that *ho* transcript levels change during the day and night in LD12:12, with a peak at ZT16, and higher levels of mRNA at ZT4 than at ZT13 (Fig. 1d). It means that *ho* expression is also cyclical in glial cells, but has a different daily pattern than in the retina.

### Effects of HO, CO, and NO on the Retina Clock

**Fig. 2:**
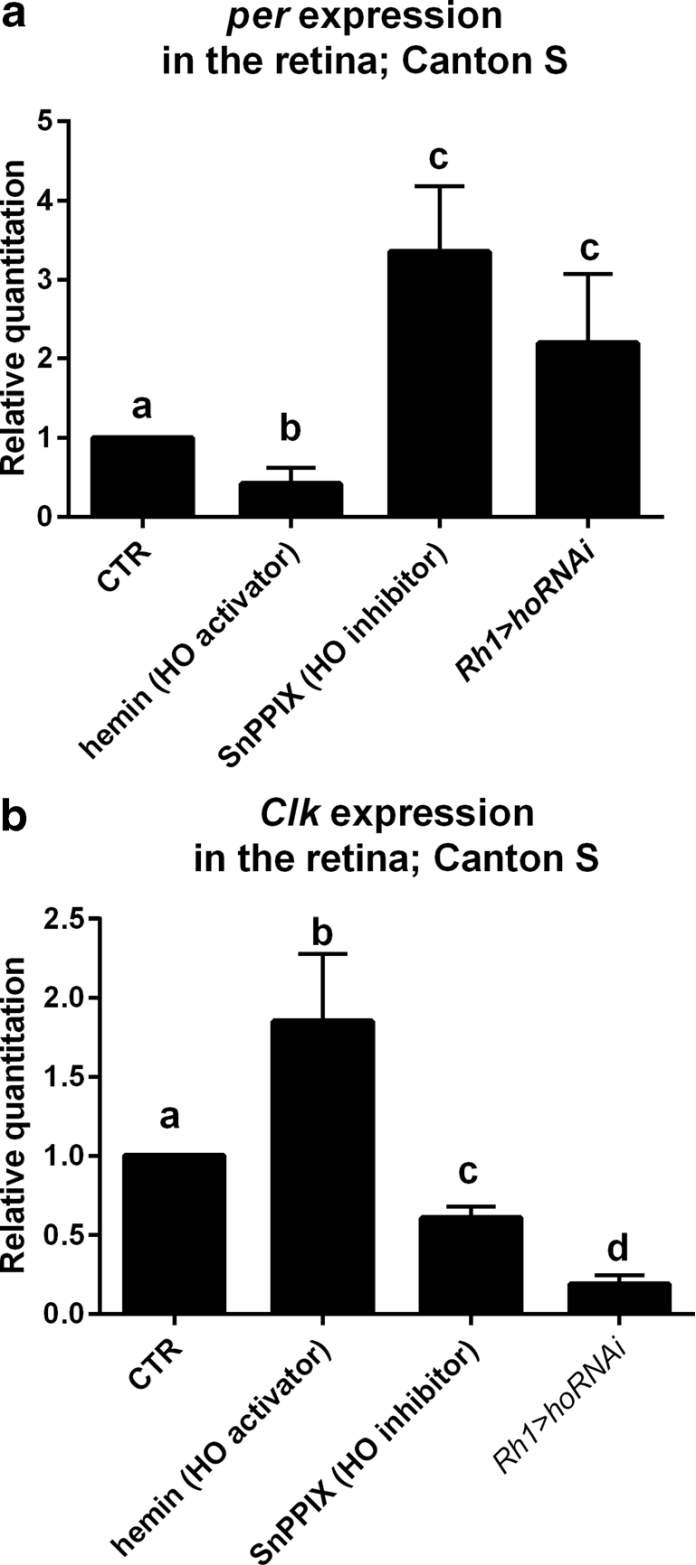
Effects of hemin (100 μM) and SnPPIX (100 μM) treatments as well as *ho* silencing in the photoreceptors (*Rh1>hoRNAi* flies) compared with the control (glucose with solvent for chemical treatment or *Rh1>Valium10* flies, respectively) on *per* (**a**) and *Clk* (**b**) gene expressions in the retina. Statistically significant differences are marked with *different letters*. Non-parametric Kruskal–Wallis test and parametric ANOVA Tukey’s test were used, *p* < 0.05; data are normalized to control (CTR = 1). *Bars* and *graphs* represent changes in mRNA levels (average of all time points ± SD)

To verify a possible effect of HO on the clock, we fed flies with an activator (hemin) or inhibitor (SnPPIX) of heme oxygenase activity. In our preliminary experiments, we have found that 100 μM hemin increases *ho* gene expression (Fig. S1), and we used this concentration for further experiments. HO activation by hemin was accompanied by a decrease in *per* expression in the retina of flies in comparison to the control flies fed with glucose (Fig. 2a), but the rhythm of *per* mRNA was maintained, with a peak at ZT16 (Fig. S2A). Surprisingly, at ZT1, *per* mRNA level was significantly higher than that in the control, while at other time points lower (Fig. S2B). In contrast, *Clk* expression was increased after hemin treatment, except at ZT4 (Fig. 2b). Moreover, in flies treated with hemin, the *Clk* mRNA oscillations were flat, and a statistically significant difference in mRNA level was detected only between ZT4 and ZT16 (Fig. S2C, D).

The effects of SnPPIX (HO inhibitor) on *per* and *Clk* expressions were opposite to those of hemin. *per* mRNA level was similar during the day, except for a trough at ZT4 (Fig. S2E). Flies fed with SnPPIX had higher *per* expression, except at ZT4 (Fig. 2a and Fig. S2F). In contrast, *Clk* mRNA level was lower after SnPPIX treatment than that in the control (Fig. 2b), except at ZT1. Moreover, the expression of *Clk* in experimental flies was the highest at the beginning of the day (ZT1), earlier than in control flies (Fig. S2G, H).

In addition to the chemical modulation of HO activity by its activator or inhibitor, we used RNAi technology in transgenic flies to decrease *ho* expression specifically in the retina. *Rh1*-GAL4 females were crossed to UAS-*ho*RNAi males to decrease HO level in photoreceptors R1–R6 which carry rhodopsin Rh1 in their offspring. As a control, we used *Rh1*-GAL4 crossed to UAS-*Valium10* flies. In the retina of experimental flies, *ho* expression was lower than that in control flies (Fig. S3A). After silencing of *ho*, the expression of *per* was higher than that in control flies at all time points studied (Fig. 2a and Fig. S3B), and the daily pattern of *per* expression was the same as that in the control (Fig. S3C). In turn, *Clk* expression was strongly decreased in the experimental flies when compared with the control (Fig. 2b and Fig. S3D), but its daily pattern was not changed (Fig. S3E).

**Fig. 3:**
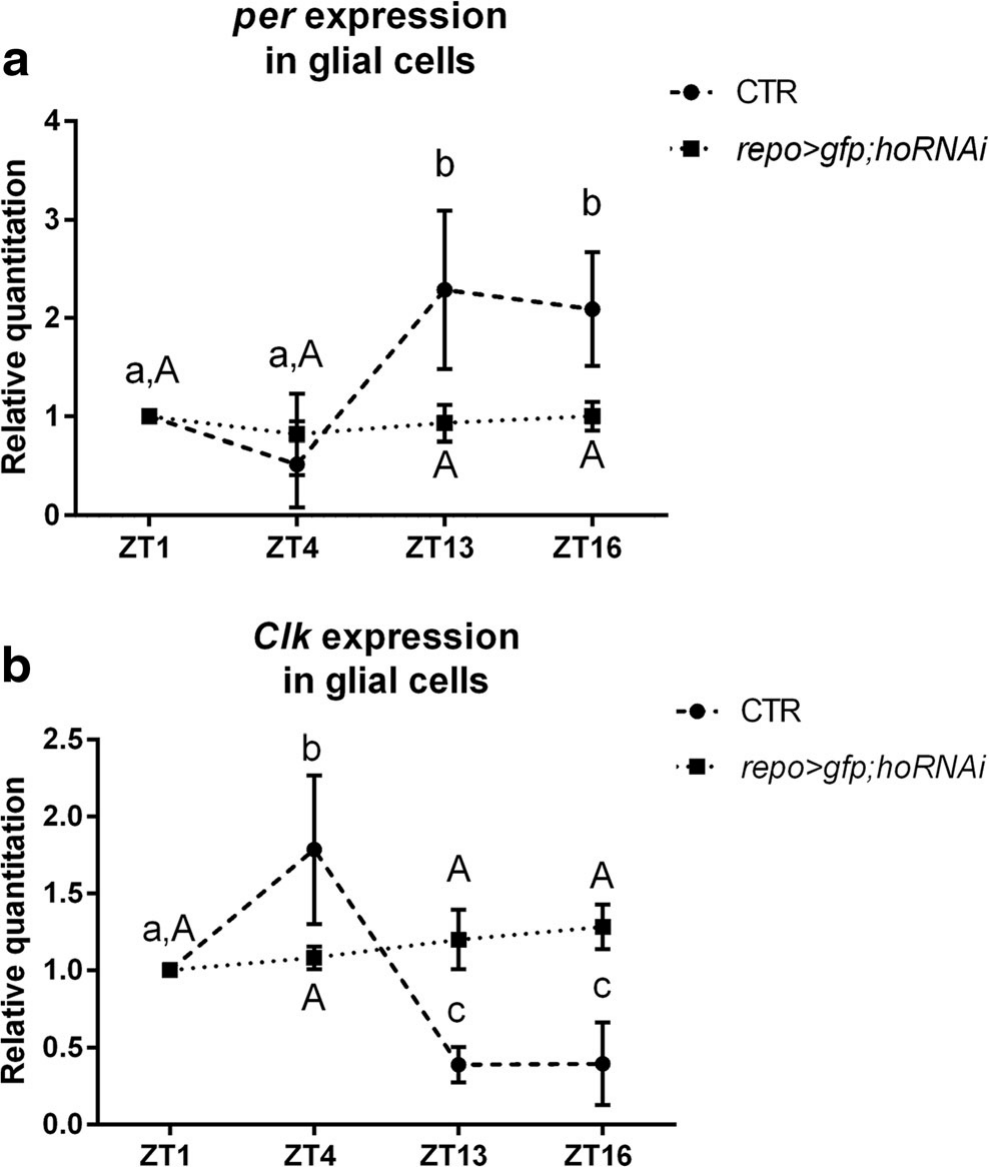
Effect of *ho* silencing in the glial cells on *per* (**a**) and *Clk* (**b**) expressions in the glial cells of *repo>hoRNAi*;*gfp Drosophila* strain. As a control, *repo>gfp* strain was used (*CTR*). Statistically significant differences are marked with *letters* (*lowercase letters* for control and *uppercase letters* for *hoRNAi*); data are normalized to ZT1 as value 1. Non-parametric Kruskal–Wallis test, parametric ANOVA Tukey’s test: *p* < 0.05. *Graph* represents average clock gene expression level ± SD

The silencing of *ho* in glial cells abolished the daily rhythm of *per* and *Clk* expressions, indicating that the clock was disrupted since in the control both mRNAs cycle during the day, with peaks at ZT16 (*per*) and ZT4 (*Clk*; Fig. 3a, b). After *ho* silencing in glial cells, *per* and *Clk* mRNA levels in those cells were the same at four time points studied (data not shown). Because the effect of *ho* silencing is different in glial cells than in the retina described above, it suggests that glial clock is different from that of the retina photoreceptors.

**Fig. 4:**
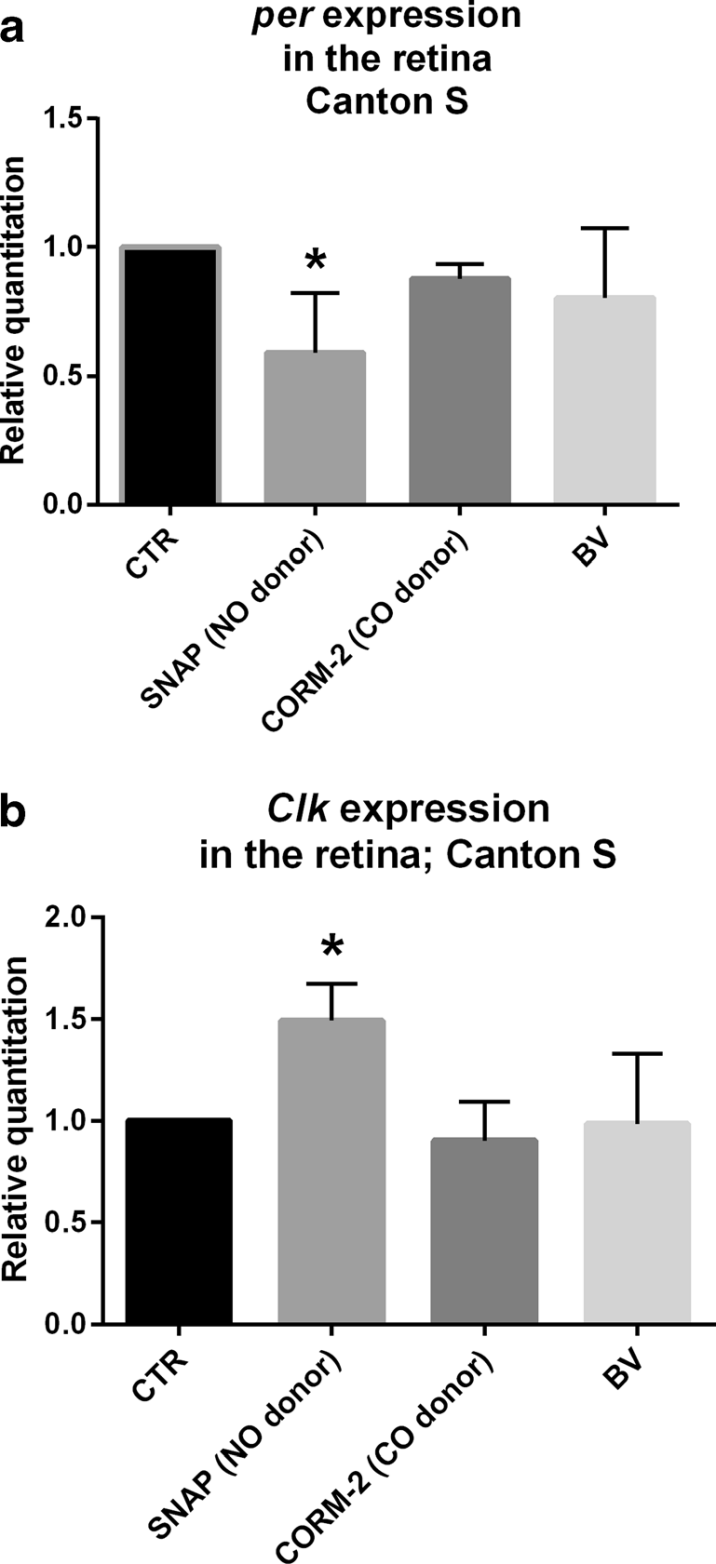
Effects of SNAP (500 μM), CORM-2 (500 μM), and biliverdin (*BV*; 500 μM) treatments compared with control on *per* (**a**) and *Clk* (**b**) gene expressions in the retina of wild-type *D. melanogaster*. Data are normalized to the control as value 1. Statistically significant differences are marked with *stars*, *p* < 0.05. Mann–Whitney test: *p* < 0.05. *Bars* represent average clock gene expression ± SD

To assess whether HO modulates the clock mechanism directly or has a rather indirect effect, we treated flies with HO activity products: CO, using CO donor CORM-2, or BV. Flies fed with 500 μM of CORM-2 as well as 500 μM or 1 mM of BV did not show any changes in *per* and *Clk* expressions (Fig. 4a, b and Fig. S4A, B), while a higher CORM-2 concentration (1 mM) was lethal for flies. We also examined the effect of NO donor because of a possible cross-talk between NO and HO [25,26]. In our preliminary study, we found that the effective concentration of SNAP, established by measuring the ROS level, was 500 μM (Fig. S5). The application of SNAP decreased *per* and increased *Clk* expressions in comparison with the control treated with a solvent (Fig. 4a, b). This effect was observed at each time point studied and was similar to that obtained after feeding flies with hemin (Fig. S6A, B).

### Effects of HO and NO on Clock Gene Expression in the Retina of *Clk*^*Jrk*^ Mutant Flies

**Fig. 5:**
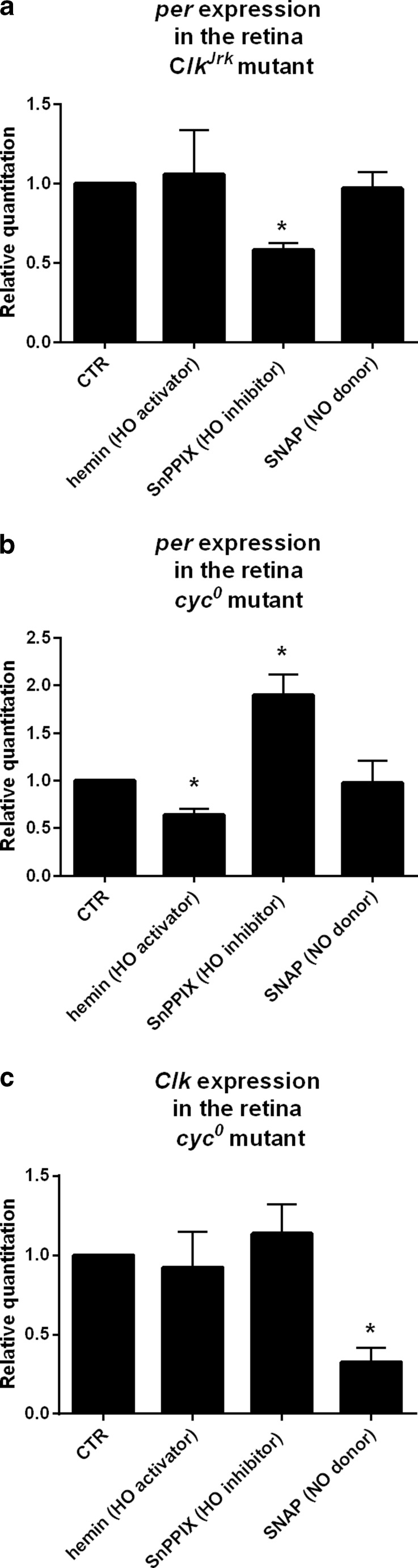
Effects of hemin (100 μM), SnPPIX (100 μM), and SNAP (500 μM) treatments compared with control on *per* (**a**, **b**) and *Clk* (**c**) gene expressions in the retina of *Clk* ^*Jrk*^ (**a**) and *cyc* ^*0*^ (**b**, **c**) mutants. Data are normalized to the control (*CTR*) as value 1. Mann–Whitney test: *p* < 0.05. *Bars* represent average of mRNA level ± SD

In mammals, NO and CO bind to clock proteins acting as transcription factors and change their ability for DNA binding [27]. Looking for a similar mechanism in *Drosophila*, we compared *per* and *Clk* expressions in wild-type flies, *Clk* ^*Jrk*^ and *cyc* ^*0*^ mutants after feeding with hemin, SnPPIX, or SNAP. *Clk* ^*Jrk*^ flies fed with hemin or SNAP did not show differences in *per* expression, but surprisingly, *per* mRNA level was significantly decreased after SnPPIX treatment (Fig. 5a). On the other hand, *cyc* ^*0*^ mutant fed with hemin showed a decrease in *per* expression, whereas SnPPIX exerted an opposite effect on *per* expression (Fig. 5b). *cyc* ^*0*^ flies after SNAP treatment did not show differences in *per* mRNA level compared with the control (Fig. 5b). In contrast, *Clk* expression in *cyc* ^*0*^ mutants fed with hemin or SnPPIX was similar to that in control flies, but flies fed with SNAP had lower *Clk* mRNA level (Fig. 5c). These results indicate that changes in HO activity and an increase in NO level affect the clock through CLOCK protein. On the other hand, the observed changes in *per* expression after SnPPIX suggest another, not clock-dependent function of PER that has already been reported in the visual system of *Drosophila* [10,12].

### Effect of HO, NO, and CO on Clock Gene Expression in S2 Cells

**Fig. 6:**
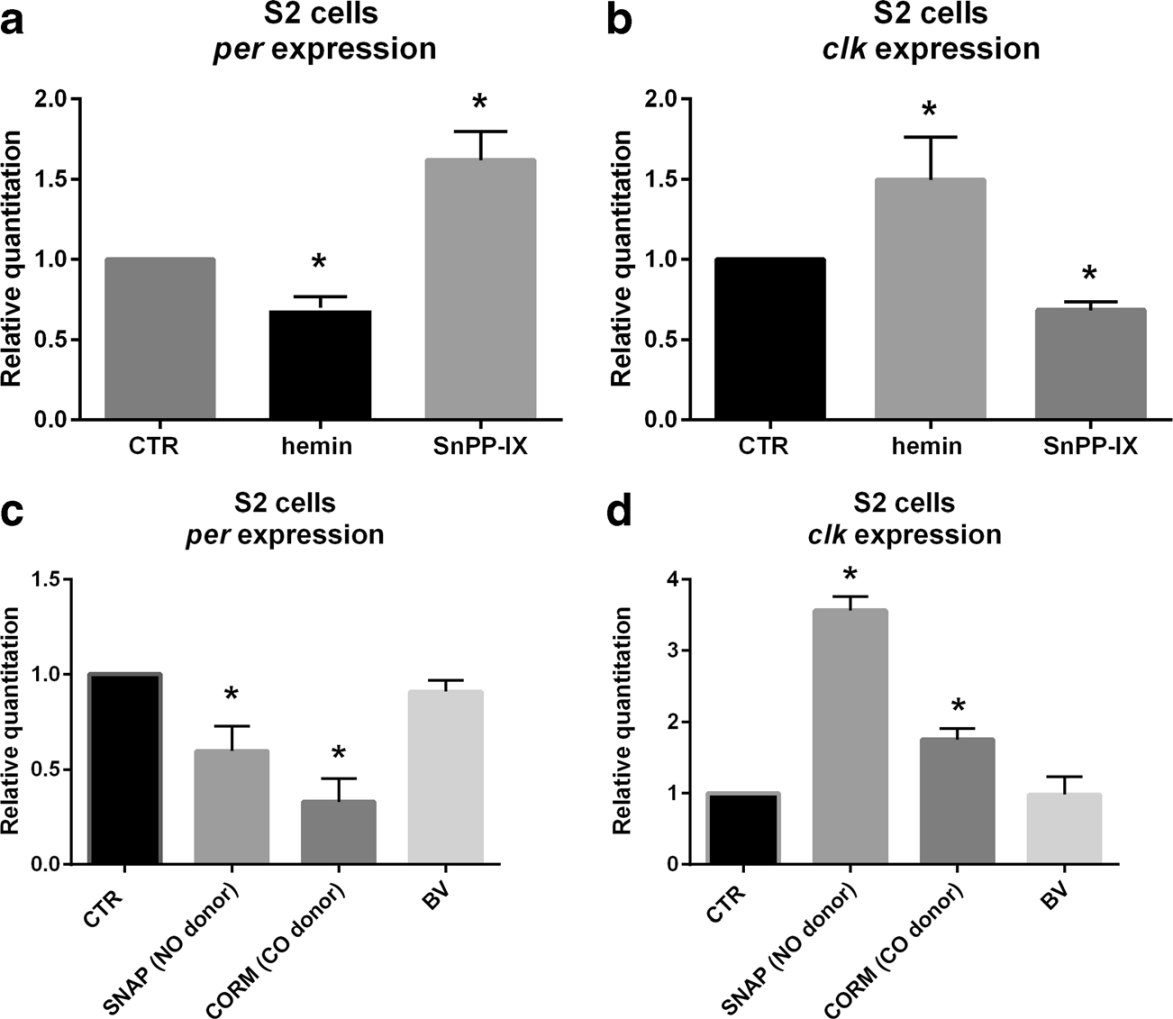
Effects of hemin, SnPPIX, CORM-2, biliverdin, and SNAP treatments on *per* (**a**, **c**) and *Clk* (**b**, **d**) gene expressions in S2 cells in vitro culture. RNA was isolated after 6 h of cell culturing with chemicals or control. Data are normalized to the control (*CTR*) as value 1. Mann–Whitney test: *p* < 0.05. *Bars* represent average clock gene expression level ± SD

In S2 cells cultured in the medium supplemented with 100 μM of hemin, *ho* expression was increased (Fig. S7A). In the case of clock genes, *per* expression was decreased and *Clk* increased after 6 h treatment with 100 μM of hemin (Fig. 6a, b); however, 50 μM of hemin had no effect on both gene expression (data not shown). In turn, 100 μM of SnPPIX increased *per* gene expression and decreased *Clk* expression after 6 h exposure to this HO inhibitor (Fig. 6a, b). NO donor, 100 μM of SNAP, decreased *per* and increased *Clk* mRNA levels (Fig. 6c, d), however did not affect *ho* expression (Fig. S7B). The effect of CORM-2 was observed after 6 h exposure to 100 μM concentration of CORM-2 (Fig. 6c, d). BV had no effect on *per* and *Clk* expressions (Fig. 6c, d).

### Effects of UV and White Light on DNA Damage in Photoreceptors

**Fig. 7:**
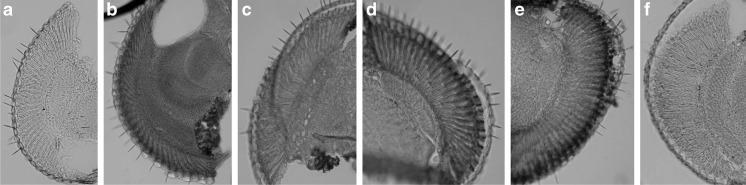
Oxidative DNA damage after UV and intense white light exposure. DNA damage is visualized as *dark color* using anti-8-hydroxyguanosine and DAB/HRP enzymatic reaction. **a** Negative control (without primary antibodies). **b** Positive control (heads incubated with cytotoxic etoposide). **c** Control flies fed with glucose, not exposed to light. **d** Flies fed with glucose, exposed to light. **e** Flies fed with HO inhibitor (SNPPIX) and exposed to light. **f** Flies fed with HO activator (hemin) and exposed to light

When flies were fed with glucose and were not exposed to UV and white light, there were almost no changes in DNA integrity (Fig. 7c), while after the exposure the HRP signal was strong in the photoreceptor nuclei (Fig. 7d). This effect was even stronger after feeding flies with the HO inhibitor (Fig. 7e). However, flies fed with the HO activator and exposed to light had less DNA damage (Fig. 7f) than the control group fed with glucose. Their level of DNA damage in photoreceptors was similar to flies which were not exposed to UV and white light (Fig. 7c).

## Discussion

### *ho* is Cyclically Expressed in the Retina and Protects Photoreceptors Against Degeneration

We showed that in the retina of *Drosophila*, the *ho* gene, encoding heme oxygenase, oscillates in expression during the day, with two peaks at the beginning of the day (ZT1) and in the middle of the night (ZT16). This rhythm is maintained in constant darkness and is abolished in the arrhythmic *per* ^*01*^ null mutant. This means that *ho* is a clock-controlled gene (ccg). The expression of *ho* is probably controlled by the autonomous clock in the retina [10,28,29] composed of circadian oscillators located in the retina photoreceptors. Their molecular mechanism is similar to that in the small LN_v_s, the main pacemaker neurons in the brain of *Drosophila* that are crucial for the circadian rhythm in locomotor activity [30,31]. However, as we showed in our previous study [10], rhythmic expression of some ccgs in the retina is also controlled by the pacemaker via the release of its two neurotransmitters, pigment-dispersing factor (PDF) and ion transport peptide. The cyclic expression of *ho* is also maintained in the brain since in whole head homogenates of *yw* wild-type flies, the mRNA of *ho* peaks at ZT8 and ZT20 [9].

In mammals, as has been found in the SCN of hamsters, heme oxygenase is more active during the night (ZT16) than during the day (ZT4) in LD12:12; the same pattern is maintained in DD [5]. We suppose that in the retina of *Drosophila*, the high expression of *ho* at the beginning of the day might be important for light adaptation of the photoreceptors and/or their protection during night/day transition. It has been reported that in mammals, *ho* expression is induced by ROS as well as by light pulse [32,33]. Since phototransduction increases the level of free radicals, this may cause cell damage and retina degeneration [15]. In addition to its role during the night/day transition, HO at ZT1 may also stimulate *Clk* expression that is the highest at ZT4, affecting the clock. The second peak in *ho* expression, at ZT16, may be connected with the regulation of both *per* and *Clk* expressions since the activation of HO by hemin at ZT16 strongly suppresses *per* expression, while *Clk* mRNA level is almost doubled than at the other time points during the day. The expression of *ho* is also cyclic in glial cells, with a peak at ZT16, so HO seems to participate in the regulation of cyclic processes in various cell types.

In the case of the retina photoreceptors, we found that HO protects them against ROS-induced degeneration, and when the HO level is low and photoreceptors are exposed to UV light, the DNA is seriously damaged.

### HO Influences the Molecular Clock

It has already been reported in mammals that heme metabolism may modulate the circadian clock since it can synchronize clock gene expression in vitro [6]. In addition, heme injections increase *mPer1* and decrease *mPer2* clock gene expression in the mouse liver [6]. Heme can also bind to PER2 protein and induce its degradation [7]. This effect is tissue-specific since heme damps PER2 rhythm in the SCN, but not in other tissues as the thymus, esophagus, or spleen [8]. Moreover, oxidative stress reduces *per* cycling, with a weak effect in clock neurons and strong in peripheral oscillators, and decreases PDF level in the projections from LN_v_s [34].

We found that the retina and glial peripheral clocks of *Drosophila* are also HO-dependent since transcription of clock main genes is affected by feeding flies with the heme oxygenase activator (hemin) or inhibitor (SnPPIX). In the pacemaker neurons of *Drosophila*, *per* and *Clk* genes are expressed in anti-phase and regulate each other’s transcription (for a review, see [13]). In the retina, *per* and *Clk* mRNAs peak at ZT16 and ZT4, respectively. Hemin, which increases *ho* expression and HO activity, had dose-dependent effects on the expressions of *per* and *Clk*. It decreased *per* mRNA at each time point studied, except at ZT1, and increased *Clk* mRNA, except at ZT4 and ZT13.

Moreover, the effects of chemical and genetic inhibition of *ho* expression were opposite on both genes’ transcriptions, increasing *per* and decreasing *Clk* mRNA levels. The opposite effects of the activator and inhibitor on *per* and *Clk* transcriptions at specific times during the day are not dependent on light, which could, for example, degrade those chemicals in food, because we obtained the same results in flies kept in DD (data not shown). This phenomenon probably results from changes of endogenous HO during the day. The high level of *Clk* mRNA at ZT1 after both hemin and SnPPIX is followed by the low level of *per* mRNA after applying both chemicals at ZT4. Moreover, *per* expression after hemin treatment was still rhythmic, with the same pattern of the rhythm, but with low amplitude. This indicates that HO damps *per* mRNA rhythm. In the case of *Clk*, the daily pattern of its expression was changed after feeding flies with hemin since the peak at ZT4 was abolished. It suggests that HO damps also *Clk* mRNA rhythm, but only at ZT4, while *per* mRNA is suppressed at the end of the night.

The obtained results indicate that HO regulates the molecular clock in the retina and in glial cells. Heme oxygenase decreases *per* expression and regulates the amplitude of *Clk* oscillations. The peak of *ho* mRNA level is correlated with the maximum of *per* mRNA and seems to be crucial for regulating the clock mechanism. The high HO level at ZT16 inhibits *per* expression and decreases PER protein level after a few hours, at the end of the night. In the case of glial cells, the lack of HO abolishes *per* and *Clk* cyclic expressions, so the clock does not function in those cells.

The mechanism of heme oxygenase action on the molecular clock may be direct by binding to clock protein or transcription factors or indirectly by products of its activity—CO, iron ions, or biliverdin. One might suppose that heme oxygenase influences the clock by regulating nitric oxide synthase activity or NO has an independent effect on the clock, as in the case of mammals [27].

The activity of clock proteins in mammals is regulated by binding CO or NO. It has been reported that the transcription factor NPAS2 can bind CO [35], while CLOCK can bind CO and NO [27]. Our results obtained using NO donor are similar to those after feeding flies with hemin since *per* mRNA level was decreased and *Clk* expression was increased. In turn, feeding flies with CORM-2 did not show that CO is involved in the clock regulation. However, in S2 cells, we observed the inhibitory effect of CORM-2 on *per* and the stimulatory effect on *Clk* expression. It may indicate that, in our experimental settings, feeding with CORM-2 was not sufficient to obtain the adequate concentration able to influence the clock mechanism in the retina. Moreover, CO action might be short-term. The effect of CORM-2 application was observed after 6 h, but not after 12 h in S2 cell culture. We did not observe any impact of biliverdin, another HO activity product, on *per* and *Clk* transcriptions.

Using arrhythmic clock mutants *Clk* ^*Jrk*^ and *cyc* ^*0*^, which do not synthesize CLK and CYC transcription factors, respectively, we found that in *Clk* ^*Jrk*^ mutant fed with hemin or SNAP, there was no change of *per* expression, while SnPPIX treatment decreased *per* mRNA level. It means that CLK is involved in HO effect on *per* expression; however, HO may also activate other transcription factors which regulate the expression of *per*, and this process is NO-independent. In turn, in *cyc* ^*0*^ mutant, the effects of hemin or SnPPIX treatment on *per* expression were similar to those observed in wild-type flies; however, no effect was found after SNAP feeding. This suggests that CLK, but not CYC, is needed for the HO-dependent process of regulating *per* expression, but it does not involve NO.

## Conclusions

HO seems to be a sensor of cell metabolism, and changes in HO activity affect the molecular mechanism of the circadian clock. This protein is important for cell survival, protecting cells against ROS-induced DNA damage and may suppress the clock under stressful conditions. HO increases the expression of *Clk*, and this process seems to depend on the CO and NO levels. NO increases *Clk* expression, but may also decrease it when the CYC protein is missing. In turn, the expression of *per* is decreased after the activation of HO. The expression of *per* may be additionally regulated by HO in a CLK-independent pathway, or *per* may have in the *Drosophila* visual system another function regulated by HO. HO seems to regulate the clock at the beginning of the day and at the end of the night by different processes.

## Electronic Supplementary Material

Below is the link to the electronic supplementary material.

Fig. S1 Effect of different concentrations of hemin (0 μM = CTR, 30, 50, 100 μM) on *ho* gene expression in the retina. Statistically significant difference is marked with *star*, *p* < 0.05. *Bars* represent average (three repetitions) ± SD of *ho* mRNA level (TIF 28 kb)

Fig. S2 Effects of hemin and SnPPIX treatment on *per* and C*lk* gene expression in the retina of wild-type *D. melanogaster* examined at four time points in LD12:12. **A**, **C**, **E**, **G**: show daily pattern of *per* or *Clk* gene expression after hemin or SnPPIX treatment; data are normalized to ZT1 as value 1. Statistically significant differences are marked with *different letters*. Non-parametric Kruskal–Wallis test, parametric ANOVA Tukey’s test, *p* < 0.05. **B**, **D**, **F**, **H** show changes in *per* and *Clk* levels compared to control (flies fed with glucose only) at every time point. Statistically significant differences are marked with *stars*. Mann–Whitney test, *p* < 0.05; data are normalized to the control at each time point, CTR as value 1. *Bars* and *graphs* represent changes in mRNA level (average ± SD), three repetitions, >30 flies each repetition (TIF 111 kb)

Fig. S3 **A** Effect of silencing *ho* gene expression measured in the retina of *Rh1>hoRNAi* flies. Silencing efficiency measured as *ho* gene expression level. Statistically significant difference is marked with *star*, *p* < 0.05. *Bars* represent average (three repetitions) ± SD of *ho* gene expression level. **B**–**E** Effects of *ho* silencing in the retinal photoreceptors R1–R6 expressing rhodopsin 1 on *per* and *Clk* gene expression in the retina of *Rh1>hoRNAi Drosophila* strain. As a control *Rh1>Valium10* strain (CTR) was used. **B**, **D** show changes in *per* and *Clk* levels compared to control at every time point. Statistically significant differences are marked with *stars*. Mann–Whitney test, *p* < 0.05; data are normalized to the control at each time point, CTR as value 1. **C**, **E** show daily pattern of *per* or *Clk* gene expression after *ho* silencing. Statistically significant differences are marked with *different letters*. Non-parametric Kruskal–Wallis test, parametric ANOVA Tukey’s test, *p* < 0.05; data are normalized to ZT1 as value 1. *Bars* and *graphs* represent mRNA level changes (average ± SD) during the day. Three repetitions, 30 flies each (TIF 62 kb)

Fig. S4 Effect of CORM-2 (**A**) and BV (**B**) on *per* and *Clk* expression level in the retina at different time points. There are no statistically significant differences. Mann–Whitney test, *p* < 0.05; data are normalized to the control at each time point, CTR = 1. *Bars* represent average of mRNA level ± SD (TIF 121 kb)

Fig. S5 Effect of SNAP feeding on ROS level in the brain. Flies fed with glucose (*blue line*, control flies) or fed with 100 μM (*green line*), 250 μM (*black line*), or 500 μM SNAP (*red line*) were decapitated, then brain cells were incubated with ROS detection reagent. *X*-axis shows fluorescence intensity, *Y*-axis shows number of counts. Shifts of curves after feeding flies with SNAP from the control one (glucose) indicate higher levels of ROS than in the control (JPG 324 kb)

Fig. S6 Effect of SNAP treatment comparing with control on *per* (**A**) and *Clk* (**B**) gene expression in the retina of wild-type *D. melanogaster*. Data are normalized to the control as value 1 at each time point. Statistically significant differences are marked with *stars*, *p* < 0.05. Mann–Whitney test; *p* < 0.05. *Bars* represent average clock genes expression ± SD (TIF 44 kb)

Fig. S7 Effect of hemin and SNAP treatments on *ho* gene expression in S2 cells. *ho* expression is increased after 100 μM hemin after both 6 and 12 h exposures. There is no effect after 50 μM hemin and SNAP treatments. Statistically significant differences are marked with *stars*, *p* < 0.05. *Bars* represent average (three repetitions) ± SD of *ho* gene expression level (TIF 88 kb)
