# Supplementary figures and images for: Interactions Between the Circadian Clock and Heme Oxygenase in the Retina of Drosophila melanogaster
Source: Mol Neurobiol. 2016 Aug 13;54(7):4953–62. doi: 10.1007/s12035-016-0026-9 (PMC5533861; doi:10.1007/s12035-016-0026-9)

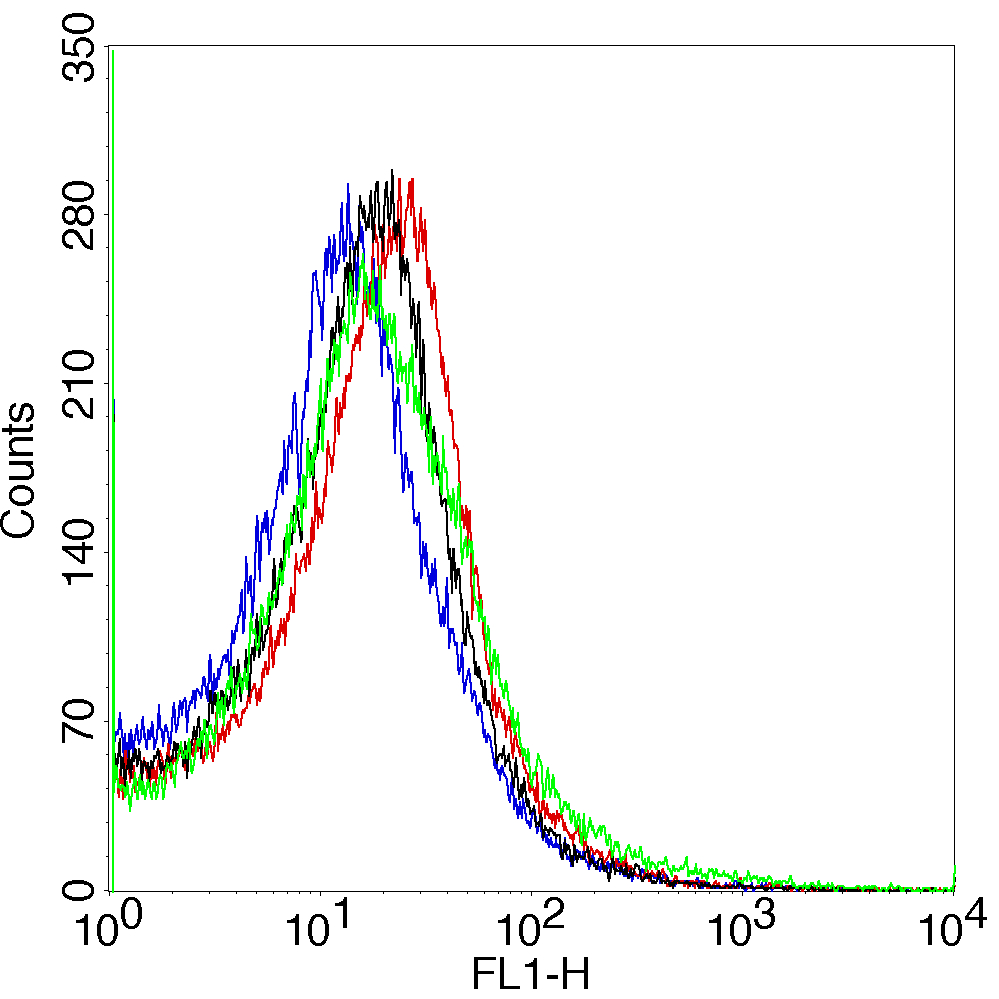

Supplement: Supplementary file 5 — Effect of SNAP feeding on ROS level in the brain. Flies fed with glucose (blue line, control flies) or fed with 100 μM (green line), 250 μM (black line), or 500 μM SNAP (red line) were decapitated, then brain cells were incubated with ROS detection reagent. X-axis shows fluorescence intensity, Y-axis shows number of counts. Shifts of curves after feeding flies with SNAP from the control one (glucose) indicate higher levels of ROS than in the control (JPG 324 kb) [file 12035_2016_26_MOESM5_ESM.jpg]
